# Pathogen and Circadian Controlled 1 (PCC1) Protein Is Anchored to the Plasma Membrane and Interacts with Subunit 5 of COP9 Signalosome in *Arabidopsis*

**DOI:** 10.1371/journal.pone.0087216

**Published:** 2014-01-27

**Authors:** Ricardo Mir, José León

**Affiliations:** Instituto de Biología Molecular y Celular de Plantas, Consejo Superior de Investigaciones Científicas-Universidad Politécnica de Valencia, Valencia, Spain; National Taiwan University, Taiwan

## Abstract

The *Pathogen and Circadian Controlled 1* (*PCC1*) gene, previously identified and further characterized as involved in defense to pathogens and stress-induced flowering, codes for an 81-amino acid protein with a cysteine-rich C-terminal domain. This domain is essential for homodimerization and anchoring to the plasma membrane. Transgenic plants with the ß*-glucuronidase* (*GUS*) reporter gene under the control of 1.1 kb promoter sequence of *PCC1* gene display a dual pattern of expression. At early post-germination, *PCC1* is expressed only in the root vasculature and in the stomata guard cells of cotyledons. During the transition from vegetative to reproductive development, *PCC1* is strongly expressed in the vascular tissue of petioles and basal part of the leaf, and it further spreads to the whole limb in fully expanded leaves. This developmental pattern of expression together with the late flowering phenotype of long-day grown RNA interference (iPCC1) plants with reduced *PCC1* expression pointed to a regulatory role of PCC1 in the photoperiod-dependent flowering pathway. iPCC1 plants are defective in light perception and signaling but are not impaired in the function of the core CO-FT module of the photoperiod-dependent pathway. The regulatory effect exerted by PCC1 on the transition to flowering as well as on other reported phenotypes might be explained by a mechanism involving the interaction with the subunit 5 of the COP9 signalosome (CSN).

## Introduction

The *Pathogen and Circadian Controlled 1* (*PCC1*) gene in Arabidopsis was originally identified as a *Pseudomonas syringae*-induced gene with a circadian controlled pattern of expression [1]. Further work revealed *PCC1* as a salicylic acid (SA)-induced gene with a potential function in controlling flowering time under UV-C light stress conditions and also under non-stressed conditions [2]. Based on bioinformatic predictions, PCC1 has been characterized as one of the so-called Cysteine-rich Transmembrane (CYSTM) domain-containing family of proteins [3]. Since plants with reduced *PCC1* expression by means of an RNAi approach (iPCC1 transgenic lines) are late flowering [2] and *PCC1* gene expression is potentially controlled by the circadian clock [1], it seems likely that PCC1 is connected to light signaling. In plants, development from seed germination to the reproductive stage is tightly controlled by light. Light perception and downstream signaling functionally interacts with different hormone signaling pathways in controlling most of the developmental transitions [4]. Gibberellins (GAs) are key hormones in many light-driven transitions [5] including seed germination [6], hypocotyl elongation during skotomorphogenesis [7], [8] and flowering time [9]. The control exerted by the combined stimuli of light and GAs on diverse developmental processes is based on multiple regulatory levels from gene transcription [10] to post-transcriptional [11] and post-translational [12] processes. In the model plant *Arabidopsis thaliana*, transcription factors of the bHLH family are key transcriptional regulators in controlling the elongation of hypocotyls in close connection with repressor proteins of the GA-related DELLA family [7], [8]. It is also well documented that DELLA proteins are substrates for the post-translational modification based on ubiquitination of lysine residues mediated by the E3 ubiquitin ligase SCF^SLY1^ complex [13], [14]. The ubiquitinating activity of the SCF complexes is dependent on the function of their four components (RBX1, Skp1-like, Cullin and F-box-proteins). Moreover, cullin must be post-translationally modified by binding the RUB/NEDD8 ubiquitin-like protein for SCF complexes to be assembled and fully active [15]. This ubiquitination machinery is negatively controlled by the function of an eight-subunit protein complex so-called COP9 signalosome (CSN), which has RUB isopeptidase activity that removes RUB modification from cullin proteins [16]. The derubylation reaction is mediated by the subunit CSN5, a zinc metalloprotease, although defective function of any of the subunits make CSN unable in derubylation [17] and cause severe developmental [18] and defense responses [19].

Here we present multiple lines of experimental evidence of the plasma membrane localization for PCC1 protein and its homodimerization. We found that the cysteine-rich C-terminus is responsible for both, anchoring to the plasma membrane and dimerization. *PCC1* gene displays a changeable pattern of expression throughout development, which is consistent with potential regulatory roles in both development and defense. Besides, PCC1 interacts with subunits 5a and 5b of CSN at the plasma membrane, which could explain the wide range of altered phenotypes observed in plants with altered *PCC1* transcript levels.

## Materials and Methods

### Plant Material and Growth Conditions

*Arabidopsis thaliana* seeds of wild type Col-0, photoreceptor mutants *phyA*, *phyB* (kindly donated by Miguel Blázquez, IBMCP, Valencia, Spain), *cry1*, *cry2* and *cry1cry2* (kindly donated by Jose Jarillo, CBGP, Madrid, Spain), *co-10* mutants and dexametasone-induced overexpression lines oxCO-GR (kindly donated by Federico Valverde, IBVF, Sevilla, Spain), or transgenic lines expressing RNAi constructs for *PCC1* gene previously described [2] were surface sterilized with 30% bleach and 0.01% Tween 20, washed extensively with miliQ sterile water, and sown in Murashige and Skoog medium supplemented with 0.8% agar and 1% sucrose. After 3 d of stratification at 4°C on Petri MS-containing plates, seeds were transferred to a growth chamber under white fluorescent white light (fluence rate of 70 µmol m^−2^ s^−1^) with a 16 h-light/8 h-dark photoperiod and a controlled temperature of 19–23°C. *Nicotiana benthamina* seeds were sown in soil and grown in green-house under a 16-hlight/ 8-h-dark photoperiod and a controlled temperature of 19–23°C.

### Quantitative RT-PCR Analysis and GUS Staining

To quantify transcript levels, total RNAs from wild type and iPCC1 seedlings, harvested at 12 hours after dawn, were extracted, purified with the RNeasy kit (QIAGEN), and analyzed by quantitative RT-PCR techniques as described previously [20]. Primers used for qPCR are included in Table 1. GUS staining was performed in samples harvested at 12 hours after dawn with X-Gluc in the presence of 5 mM ferrycianide/ ferrocyanide redox buffer.

**Table 1:** Oligonucleotides used for qRT-PCR in this work.

Primer name	Sequence (5′to 3′)	Reference
qACT2-D	ttgttccagccctcgtttgt	[20]
qACT2-R	tgtctcgtggattccagcag	[20]
qPCC1-2D	tgctccagcctctgtacatca	[2]
qPCC1-2R	cgacttctgtctcatcatgctga	[2]
qFT-D	caaccctcacctccgagaatat	[2]
qFT-R	tgccaaaggttgttccagttgt	[2]
qCO-D	aacgacataggtagtggagagaacaac	[2]
qCO-R	gcagaatctgcatggcaataca	[2]
qGID1a-F	gtgacggttagagaccgcga	[20]
qGID1a-R	tccctcgggtaaaaacgctt	[20]
qGID1b-F	ttacggtcaaggaactcggc	[20]
qGID1b-R	tcgccctgacggttctttc	[20]
qGID1c-F	cggctcaaatcttcgatctgg	[20]
qGID1c-R	ttggcatttgcagggactttc	[20]
qRGA-F	acttcgacgggtacgcagat	[20]
qRGA-R	tgtcgtcaccgtcgttcct	[20]
qGAI-3UTR-F	aatgaattgatctgttgaaccgg	[20]
qGAI-3UTR-R	ggcttcggtcggaaatctatc	[20]

### Hormone Treatments and Hypocotyl Elongation Tests

Imbibed seeds from wild type Col-0 and three independent iPCC1 transgenic lines were stratified at 4°C for 4 days, then successively transferred to white light at 21°C for 6 h, and to darkness for additional 18 h before being incubated for 4 additional days under different light qualities supplied by LED lights in a Percival growth chamber. Fluence rates of 70 µmol m^−2^ s^−1^ for white light; 5 µmol m^−2^ s^−1^ for far-red light; 30 µmol m^−2^ s^−1^ for red light and 10 µmol m^−2^ s^−1^ for blue light were used. After different light quality incubation, hypocotyls of seedlings were laid on acetate sheets, scanned and the length measured by using ImageJ software. Around 20 seedlings per genotype and condition were measured in each of the three replicate experiments performed and the mean value calculated. The results are expressed as the mean of three replicates ± SD. When indicated the active gibberellin GA3 or the gibberellins biosynthesis inhibitor paclobutrazol (PAC) were added to the growth media at the indicated concentrations. Treatments with SA were performed by adding the indicated concentrations to liquid media supplemented with 0.02% Tween-20 as surfactant.

### Transient and Stable Transformation of Nicotiana and Arabidopsis with GFP-tagged Versions of PCC1

The PCC1 coding sequence was mobilized from entry Gateway plasmids by recombination to destination binary pGWB5 and pGWB6 vectors [21] to generate constructs for C- and N-terminal PCC1 fusion to GFP. A control GFP-stop-PCC1 construct expressing free GFP and a GFP-Δ177PCC1 construct expressing a truncated version containing the first 177 nucleotides and excluding the 3′-end coding for the C-terminus were also mobilized from entry vectors to the corresponding destination binary vectors using Gateway technology. pGWB5 was also used to generate a GFP-tagged CSN5B subunit of COP9 signalosome. Nicotiana leaves were transiently transformed by infiltration with *Agrobacterium tumefaciens* C58 strain co-transformed (1:1) with the corresponding plasmids and the P19 suppressor of silencing. Plasmids expressing the whole PCC1 protein with C-terminal fusions to GFP were also used to stably transformed *Arabidopsis thaliana* by floral dipping in suspensions of *Agrobacterium tumefaciens* C58 strain transformed with the corresponding plasmids. Primary transformants were selected in kanamycin-supplemented media and homozygous T3 seeds were used throughout this work.

### Yeast Two-Hybrid (Y2H) Screening of PCC1 Interactors

A truncated version of PCC1 containing the first 59 amino acids was cloned into pB66 as a C-terminal fusion to Gal4 DNA-binding domain (N-Gal4-PCC1(1-59)-C) and used as a bait to screen a random-primed 7-day old *A. thaliana* seedlings cDNA library containing 98.8 million cDNA clones cloned into pP6 vector. pB66 and pP6 are derived from pAS2ΔΔ and pGADGH plasmids, respectively [22]. Screening was performed by using a mating approach with Y187 (matα) and CG1945 (mata) yeast strains as previously described [22]. A total of 31 His+ colonies were selected on minimal medium lacking tryptophan, leucine and histidine. The prey fragments of the positive clones were amplified by PCR and sequenced at their 5′ and 3′ junctions. The resulting sequences were used to identify the corresponding interacting proteins in the GenBank database (NCBI).

### Protein-Protein Interaction Tests Based on Y2H, BiFC and IP-based Pull-down

Protein interactions were tested in yeast by subcloning baits and preys fused to the DNA binding (DB) and activation (AD) domains of GAL4 and the reverse option in pDBLeu and pPC86 vectors (Invitrogen). Selection was performed by plating serial dilutions of transformed yeasts in minimal medium –Trp-Leu-His supplemented with increasing concentrations of 3-aminotriazole. In planta interactions between proteins were tested by Bifunctional Fluorescence Complementation (BiFC) trough transient co-transformation of Nicotiana leaves by agroinfiltration with pair of proteins each fused to half of the YFP molecule in pYFC43 and pYFN43 vectors [23]. Reconstitution of YFP-based fluorescence was visualized by a TCS SL Leica confocal microscope. Finally, versions of PCC1 fused to GFP, HA and c-myc tags and GFP-tagged versions of GFP-Δ177PCC1 and CSN5B subunit of COP9 signalosome were co-transformed in Nicotiana leaves in the presence of the P19 suppressor of silencing. Total protein extracts were obtained by grinding leaf tissue in liquid nitrogen and extraction with TBS buffer supplemented with protease inhibitor cocktail (Sigma) and detergent as indicated. Immunoprecipitation of total protein extracts was performed with magnetic beads covered with polyclonal rabbit antibodies against HA or GFP tags (Milteny). Pulled-down proteins were further analyzed by Western blot with primary antibodies against HA (polyclonal from Abcam), GFP (monoclonal from Clontech) or c-myc (polyclonal coupled to HRP from Sigma) and secondary anti-rabbit or anti-mouse antibodies coupled to horseradish peroxidase (GE Healthcare). The Enhanced Chemiluniscence (ECL) detection kit (GE Healthcare) was used to expose Fujifilm.

### Generation of Double iPCC1/35S::GR-CO Transgenic Plants and Quantification of Flowering Time

iPCC1 plants with extremely reduced levels of *PCC1* transcript because of the over-expression of an RNAi construct for PCC1 [2] were crossed to *co-2* mutant plants transformed to overexpress CO fused to the Glucocorticoid Receptor (*35S::CO-GR/co-2*) that makes CO functional in the nucleus only upon treatment with a GR ligand such as dexamethasone (DEX) [24]. A PCR-based genotyping procedure using specific primers for *CO* (5′-GCAGAATCTGCATGGCAATGGCAATACA-3′) and *PCC1* (5′-CGCTTACTCTGATGTACAGA -3′) and common primers (5'-GCTCCTACAAATGCCATCA-3') from *35S* promoter sequence was used. Flowering time was quantified by counting rosette plus cauline leaves upon bolting as previously reported [25].

## Results

### PCC1 is a Small Protein Anchored to the Plasma Membrane

**Figure 1:**
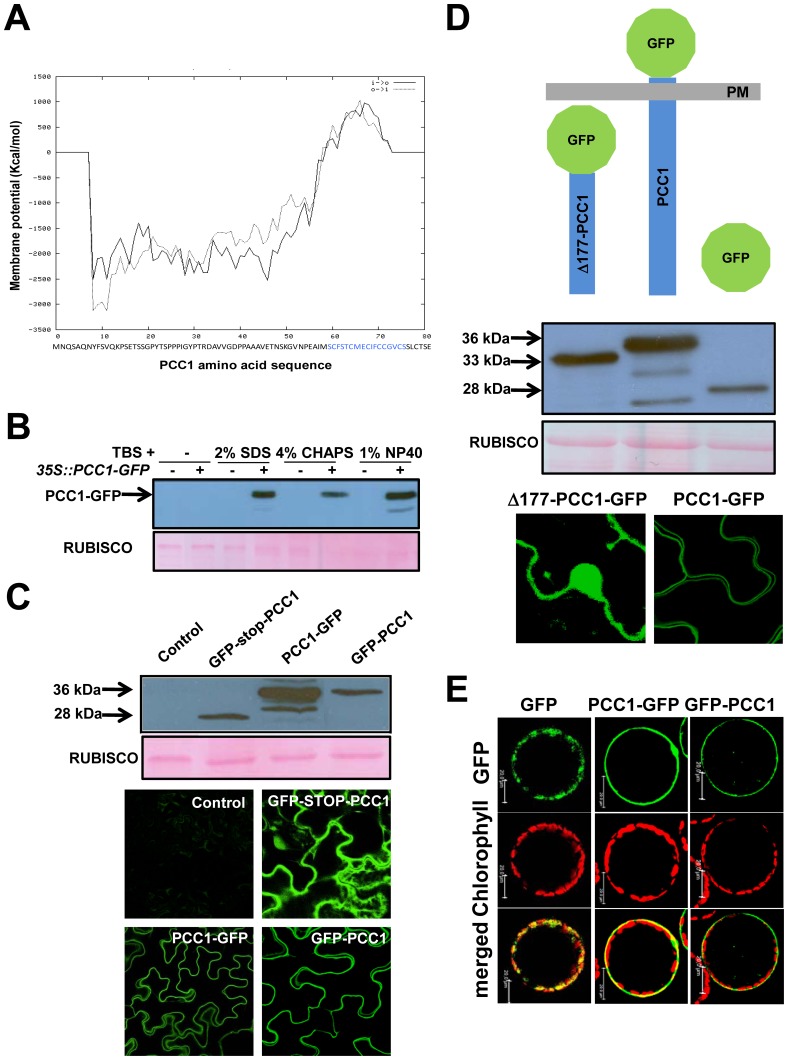
Plasma membrane localization of PCC1 protein. (A) Bioinformatic prediction of transmembrane potential for PCC1 using TMPred tool. The C-terminal domain with positive potential is written in blue in the amino acid sequence below the plot. (B) Extraction of PCC1 protein from *Nicotiana benthamina* leaves transiently transformed with *35S::PCC1-GFP* construct was assessed by using TBS buffer supplemented or not with different detergents as indicated, and further detected by Western blot with anti-GFP antibodies. Ponceau S-stained Rubisco is shown as loading control. (C) Expression of GFP-tagged versions of PCC1 in its C- (PCC1-GFP) and N-terminus (GFP-PCC1) as wells as the free GFP control (GFP-stop-PCC1) was analyzed by Western blot with anti-GFP antibodies and confocal microscopy. (D) Expression of a GFP-tagged truncated PCC1 version (Δ177-PCC1-GFP) without the potential C-terminal membrane-associated domain leads to cytoplasmic localization instead of the membrane localization for the whole GFP-PCC1 protein. (E) Isolation of protoplasts from transiently transformed *Nicotiana benthamina* leaves confirmed the plasma membrane association of PCC1 and allowed to rule out cell wall localization.

*Pathogen and Circadian Controlled 1* (*PCC1*) was originally identified as an early activated gene upon infection with the bacterial pathogen *Pseudomonas syringae AvrRpt2* that shows an expression pattern controlled by the circadian clock [1]. Later on *PCC1* was identified in a comparative transcriptomic analysis of SA-deficient versus wild type plants as a SA- and UV-C light-induced gene that codes for a potential activator of stress-stimulated flowering in Arabidopsis [2]. *PCC1* is a small gene that codes for an 81 amino acids protein with a molecular mass of 8.4 kDa. Despite its low hydrophobicity, estimated by its GRAVY index [26] of –0.129, much lower that the one observed for most of the membrane proteins so far reported, PCC1 has been identified in two previous reports searching for plasma membrane associated proteins in Arabidopsis [27], [28]. Further *in silico* analysis of PCC1 sequence with the membrane specific TMPred tool [29] predicts a transmembrane helix in the C-terminus (between amino acids 60 and 78) of the protein (Fig. 1A), which coincides with its most hydrophobic region according to its GRAVY index value, which is slightly over 1.5 between the amino acids 65 and 75. However, the use of different bioinformatic tools for the prediction of the cellular localization of proteins yields unequal results from extracellular to cytoplasmic, nuclear or even chloroplastic localization. This discrepancy in the predictions prompted us to check the subcellular localization of PCC1 through several molecular experimental approaches. First, we generated constructs to transiently express recombinant versions of PCC1 protein fused in its N- and C-terminus to GFP under the 35S promoter in *Nicotiana benthamiana*. Figure 1B shows the levels of PCC1-GFP protein extracted with either TBS buffer or TBS buffer supplemented with different detergents. Only in the presence of detergents, PCC1-GFP protein was efficiently extracted as demonstrated by Western blot using anti-GFP antibody (Fig. 1B). A confocal microscopy analysis of Nicotiana leaves transiently transformed with the 36 kDa fusion proteins PCC1-GFP, GFP-PCC1 and a control GFP-stop-PCC1 construct, which expresses free 28 kDa GFP, pointed to fluorescence associated to the plasma membrane for both PCC1-GFP and GFP-PCC1 fusion proteins, in contrast to the localization of free GFP in the cytosol and nucleus (Fig. 1C). To demonstrate that the membrane localization was dependent on the hydrophobic C-terminus region being anchored to the plasma membrane, we generated a C-terminal fusion to GFP of the truncated version GFP-Δ177PCC1 expressing the first 177 bp of the coding sequence and thus lacking the C-terminus. We first checked that the different constructs expressed proteins of 36 kDa, 33 kDa and 28 kDa corresponding to GFP fusions to the full PCC1 protein, the truncated version and free GFP, respectively (Fig. 1D). The typical fluorescence associated to the plasma membrane of the PCC1-GFP protein was changed to cytoplasmic and nuclear localization for the truncated version lacking the C-terminus (Fig. 1D), thus confirming the essential role for the C-terminus to be anchored to the plasma membrane. These data were further confirmed by co-localization studies of fluorescence associated to GFP and to the membrane-specific staining with fluorophore FM4-64 in Nicotiana leaves transformed with PCC1-GFP and Δ177PCC1-GFP. We found co-localization in the plasma membrane for PCC1-GFP but not for the truncated version (Fig. S1). To check the possibility that PCC1 is associated to the cell wall, protoplasts were isolated from *Nicotiana benthamiana* transiently transformed with *35S::PCC1-GFP* and *35S::GFP-PCC1* constructs as mentioned above. Figure 1E shows that fluorescence associated to GFP was located at the periphery of cell wall-free protoplast, thus confirming that PCC1 is neither located in the cell wall nor in the leaf apoplast. Finally, we also checked whether fluorescence associated to PCC1-GFP expression was still restricted to the plasma membrane in plasmolysed cells of Nicotiana leaves transformed with *35S::PCC1-GFP* construct. Figure S2 shows that GFP fluorescence was detected in the two plasma membranes of adjacent cells and no fluorescence was detected either in the apoplast or cell walls of plasmolysed cells.

**Figure 2:**
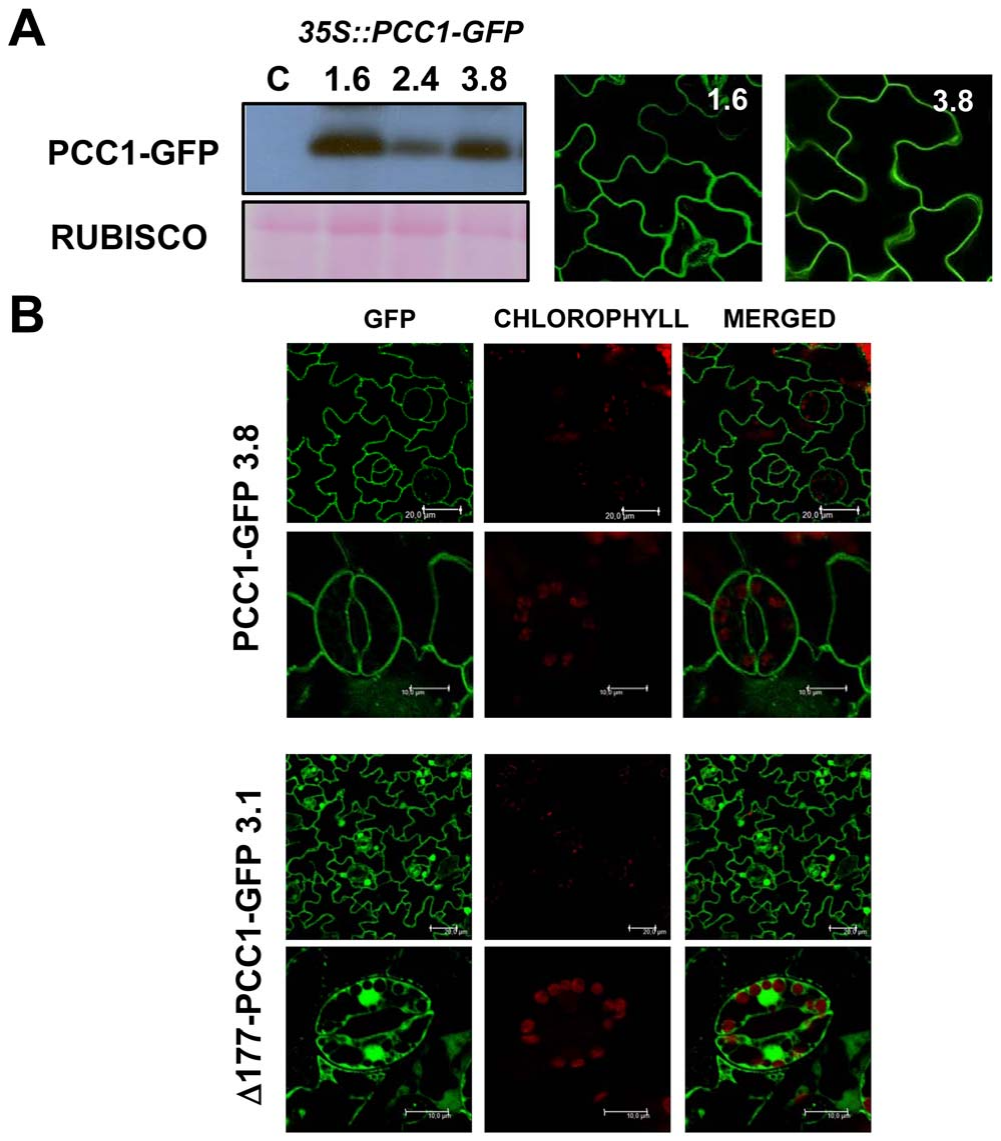
Plasma membrane localization of PCC1 in transgenic *35S::PCC1-GFP* Arabidopsis plants. (A) Levels of PCC1-GFP protein in three independent homozygous lines and control C non-transformed plants were analyzed by Western blot with anti-GFP antibodies. PonceauS-stained Rubisco is shown as loading control. Transgenic lines 1.6 and 3.8 with maximal PCC1 expression showed GFP-associated fluorescence by confocal microscopy in the plasma membrane. (B) Membrane-associated localization of PCC1-GFP contrasts with Δ177PCC1-GFP that localizes in both the cytoplasm and nucleus. The second row of images for every genotype shows magnification of stomata guard cells.

To rule out the possibility that transient expression of Arabidopsis *PCC1* in *Nicotiana benthamiana* causes aberrant protein localization on PM, transgenic Arabidopsis lines stably expressing the *35S::PCC1-GFP* construct were generated. Figure 2A shows that independent transgenic lines overexpressing PCC1-GFP displayed a plasma membrane-associated fluorescence pattern similar to that described above in transient experiments in Nicotiana (Fig. 1). Moreover, transgenic lines expressing *35S::Δ177PCC1-GFP* construct showed fluorescence in the cytoplasm and the nuclei (Fig. 2B), thus confirming that PCC1 lacking the C-terminal domain is not anchored to the plasma membrane.

Because some algorithms predicted plastid subcellular localization for PCC1, we focused our attention on these organelles. We found that PCC1 was clearly excluded from chloroplasts in epidermal leaf cells as well as in the guard cells of stomata (Fig. S3), thus allowing to rule out a functional association of PCC1 with plastids.

### PCC1 Homodimerization and Anchoring to the Plasma Membrane require its C-terminus

**Figure 3:**
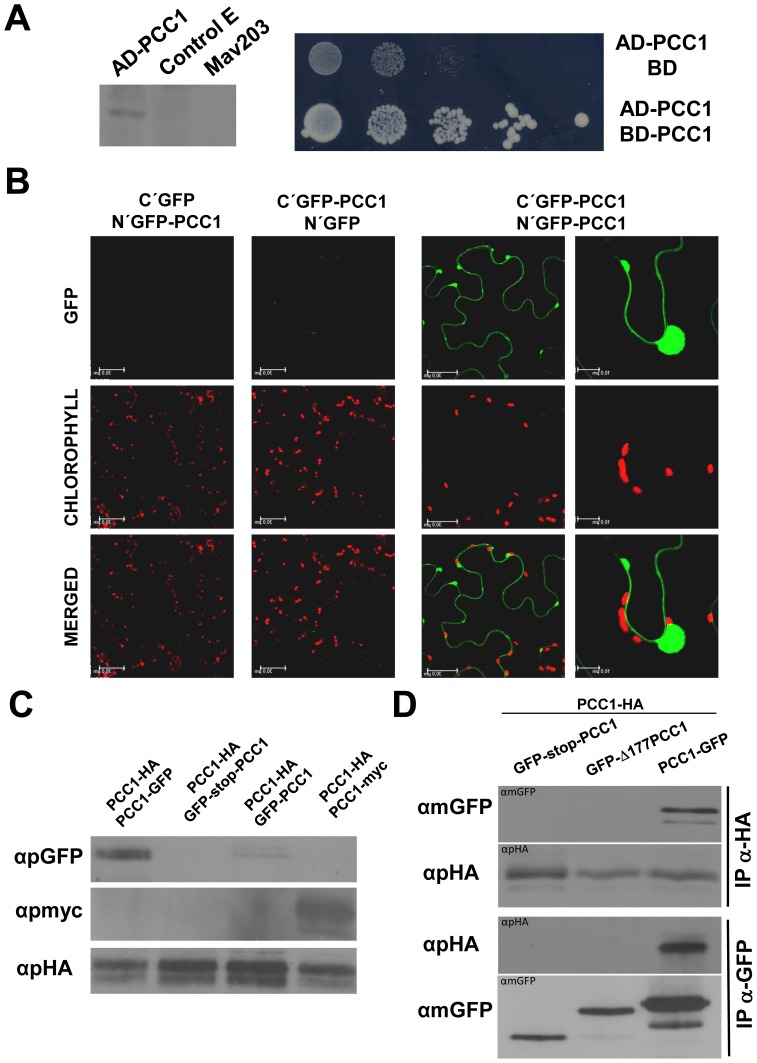
Homodimerization of PCC1. (A) The fusion of PCC1 with the activation domain (AD) of GAL4 is expressed in Mav203 yeast strain as shown by Western blot with anti-AD antibodies. A negative control E and the non-transformed yeast are also shown (top panel). Growth of yeasts co-transformed with AD-PCC1 and PCC1 fused to the DNA binding domain of GAL4 (BD-PCC1) but not with AD-PCC1 and the empty BD vector in minimal media –Leu – Trp –His is indicative of self-interaction of PCC1 in yeast two-hybrid. (B) Bimolecular fluorescence complementation (BiFC)-based demonstration of PCC1-homodimerization and localization of dimers in the plasma membrane of *Nicotiana benthamiana* leaves transiently co-transformed with the indicated constructs. (C) Confirmation of PCC1 homodimerization by pull-down assays in *Nicotiana benthamiana* leaves transiently co-transformed with PCC1-HA and GFP- and c-myc-tagged versions of PCC1 as indicated. Immunoprecipitation (IP) was performed with anti-HA and pulled-down proteins detected by Western blot with the polyclonal antibodies indicated. (D) Homodimerization of PCC1 required the transmembrane C-terminal domain as demonstrated by pull-down assays in *Nicotiana benthamiana* leaves transiently co-transformed with PCC1-HA and GFP-tagged versions of complete and truncated PCC1 molecules. IPs using anti-HA and anti-GFP followed by WB using the indicated antibodies are shown at the left.

To check whether PCC1 may interact with itself, we first conducted a yeast two hybrid (Y2H) approach. PCC1 protein fused to the activation domain (AD) of *GAL4* was expressed in Mav203 strain of *Saccharomyces cerevisiae* as demonstrated by Western blot with an antibody against the AD (Fig. 3A). Concomitantly, only yeast transformed with both AD-PCC1 and PCC1 fused to the binding domain of *GAL4* (BD-PCC1) were able to grow in His-free selective medium (Fig. 3A), thus suggesting that PCC1 interacts with itself to form dimers or higher order oligomers in yeast. To confirm the interaction *in planta* we used Bimolecular Fluorescence Complementation (BiFC) assays in *Nicotiana benthamiana*. Fluorescence was only observed when Nicotiana leaves were transformed with both ĆYFP-PCC1 and ŃYFP-PCC1 constructs leading to YFP reconstruction in the plasma membrane (Fig. 3B), thus confirming both the membrane localization and the intermolecular interaction for PCC1. Interestingly, the complemented fluorescence was detected in both plasma membrane and associated vesicle-like formations (Fig. 3B). The interaction was further confirmed by immunoprecipitation (IP)-based pull-down assays followed by Western blot. Nicotiana leaves were co-transformed with PCC1-HA and each of the following constructs expressing PCC1 fused to the indicated tags: PCC1-GFP, GFP-PCC1, GFP-stop-PCC1 and PCC1-myc. After IP with anti-HA, we detected 36 kDa PCC1-GFP and GFP-PCC1 by Western blot with anti-GFP antibody, as well as 24 kDa PCC1-myc in the corresponding immunoprecipitated proteins (Fig. 3C). As a negative control, no GFP-tagged protein was pulled down in leaves co-transformed with PCC1-HA and GFP-stop-PCC1 construct, which expresses free GFP (Fig. 3C), suggesting that interaction based on pull-down techniques was specific. By using a similar pull-down approach with leaves co-transformed with PCC1-HA and the GFP fused to the PCC1 version truncated in its C-terminus (GFP-Δ177PCC1) we further demonstrated that homodimerization of PCC1 required the cysteine-rich C-terminus of the protein. Figure 3D shows that GFP-fused protein was detected in the anti-HA-immunoprecipitated proteins from leaves transformed with PCC1-GFP but not in leaves transformed with GFP-stop-PCC1 or GFP-Δ177PCC1. The reverse IP with anti-GFP antibodies, which pulled down all three GFP, GFP-Δ177PCC1 and full size PCC1-GFP proteins, only allowed detecting the HA-tagged protein in the IP from PCC1-GFP co-transformed leaves (Fig. 3D). Together our findings demonstrate that PCC1 interacts with itself through its C-terminal domain rich in cysteine residues, which, as shown above, is also essential for being anchored to the plasma membrane.

### Developmental Pattern of *PCC1* Expression

**Figure 4:**
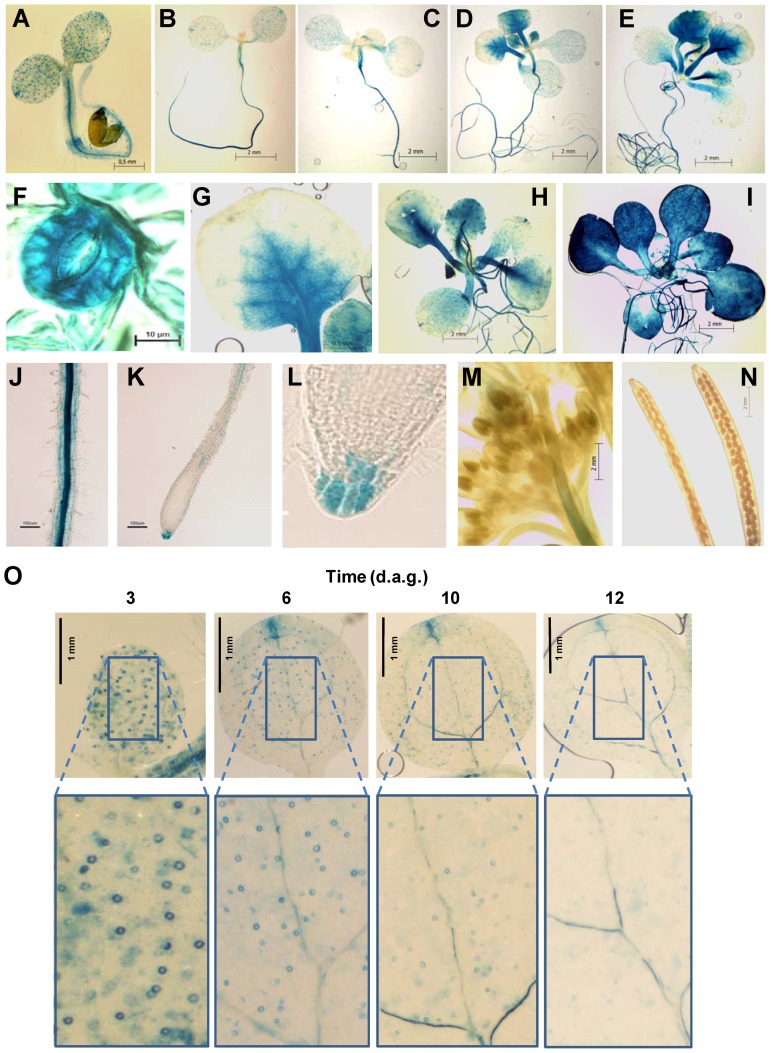
Spatial pattern of *PCC1* expression analyzed with *pPCC1::GUS* transgenic plants. GUS-stained seedlings of (A) 4 days after sowing (d.a.s.); (B) 6 d.a.s.; (C) 8 d.a.s.; (D) 10 d.a.s.; (E) 14 d.a.s. (F) Detail of GUS-stained guard cells of seedling shown in (A). (G) Leaf showing showing staining from the petiole to the distal parts. (H) and (I) Control untreated and 0.1 mM SA-treated 14-day old seedlings, respectively. (J) Vascular tissue stained in the upper part of roots. (K) Absence of GUS staining in the elongation zone and tip of roots. (L) Detail of stained calyptra. (M) and (N) Absence of expression in flower and siliques, respectively. (O) Time-course of GUS staining in cotyledons at different times after germination showing the stomata- and vascular tissue-associated patterns. The generation of *pPCC1::GUS* transgenic lines and the protocols used for GUS staining were previously reported [33].

We have previously reported that *PCC1* gene expression changes throughout post-germination development, with low levels before the transition to flowering and shifting to high levels during that developmental transition [2]. A 1.1 kb promoter sequence upstream the *PCC1* initiation codon was fused to the ß*-glucuronidase* (*GUS*) reporter gene and the resulting construct was used to transform Arabidopsis plants. We then selected three independent homozygous *p1100PCC1:GUS* transgenic lines, which were used to check the *PCC1*-directed expression in different organs and at different times after germination. In *p1100PCC1:GUS* lines, the pattern of expression was restricted to the vascular tissue in roots, hypocotyls and cotyledons, as well as to the stomata in cotyledons before the transition from vegetative to reproductive shoot apical meristem (Fig. 4A-C, F). However, *PCC1* expression decreased progressively in cotyledons as the transition to reproductive apical meristem approached (Fig. 4O), which under our experimental conditions occurs around 9 days after seed germination [2]. By 8 days after germination, lower expression was detected in stomata and vascular bundles (Fig. 4C). By day 10, GUS staining was strong in the petioles of the first pair of leaves and expanded to the basal part of leaves and subsequently to the rest of the leaf through the vascular tissue and mesophyll (Fig. 4D, G). At longer times after germination, the GUS staining was spread all over the leaf blade and vasculature (Fig. 4E). No expression was detected either in flowers (Fig. 4M), siliques (Fig. 4N) or the elongation zone of the roots (Fig. 4K). GUS staining was detected in the root cap (Fig. 4K, L). The previously characterized SA-induced pattern of *PCC1* expression [2] was also confirmed in *p1100PCC1:GUS* plants with stronger staining all over SA-treated seedling compared to untreated ones (Fig. 4H-I).

### PCC1 and the Photoperiod-Dependent Flowering Pathway

**Figure 5:**
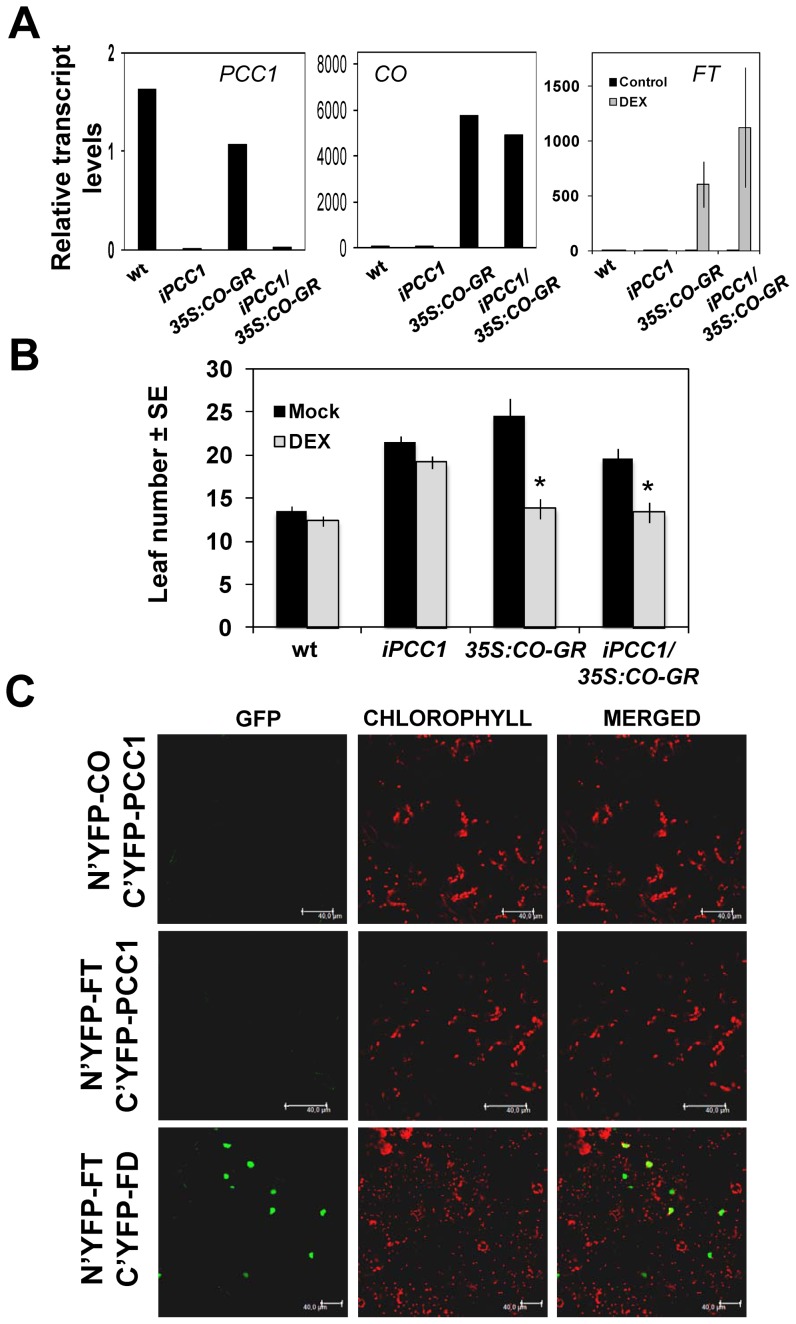
PCC1 does not interfere with photoperiod dependent floral transition pathway nor interacts with its regulatory components. (A) *PCC1*, *CO* and *FT* transcript levels in wild type, double transgenics *iPCC1/35S::CO-GR* and their parental plants in the absence and presence of 10 µM of the GR ligand dexamethasone (DEX). (B) Flowering time quantified by counting total (rosette plus cauline) leaves in long day-grown plants of the genotypes described in (A) treated or not with DEX. * represents statistically significant (p<0.05 in Students t-test) different values in DEX-treated compared to untreated (Mock) seedlings. (C) Analysis of potential interactions between PCC1 and CO or FT by BiFC. The interaction between FT and FD in nuclei is shown as positive control.

The pattern of expression observed for PCC1 during the events involved in controlling the transition to flowering under inductive long days suggests that PCC1 might be connected to or even participate in the signaling pathway controlling the photoperiod-dependent flowering. CONSTANS (CO) and FLOWERING LOCUS T (FT) are essential regulators in the photoperiod-dependent flowering pathway [30] and FT has been characterized as a mobile signal translocated from leaves through the vascular tissue to the shoot apical meristem (SAM) to activate flowering upon interaction with FD transcription factor [31], [32]. The fact that *PCC1* expression progresses from SAM to leaves and, in turn, FT is exported from leaves to SAM, might be related to several alternative hypothesis: PCC1 might interfere with CO activating *FT;* or PCC1 might physically interact with either CO or FT thus modulating their localizations/functions; or might interfere with FT translocation to SAM and its further interaction with FD transcription factor. To test the first hypothesis, iPCC1/*35S::CO-GR/co-2* plants were generated by crossing iPCC1 plants with extremely reduced levels through an RNAi approach [2] to *co-2* mutant plants transformed to overexpress CO fused to the Glucocorticoid Receptor. Because both constructs contained resistance to kanamycin as selection marker, we developed a PCR-based genotyping procedure using specific primers for *CO* and *PCC1* and common primers from *35S* promoter sequence. We confirmed by qRT-PCR that homozygous double transgenic plants overexpressed CO while showing strongly reduced *PCC1* levels (Fig. 5A). Moreover, *FT* expression was activated upon Dex treatment by inducing CO nuclear translocation (Fig. 5A). We observed that despite the strong down-regulation of *PCC1*, double transgenic plants only flowered earlier in the presence of Dex, this is, when FT was activated (Fig. 5B). These data support that PCC1 is not required for the activation of *FT* by CO and the subsequent floral transition.

To test whether PCC1 might interact *in planta* with CO or FT, BiFC was used in *Nicotiana benthamiana* leaves co-transformed with the corresponding proteins fused to N- and C- terminal parts of YFP. Neither co-transformation with CO and PCC1 or FT and PCC1 were able to reconstruct GFP fluorescence (Fig. 5C). As a positive control, the previously reported interaction of FT and FD was observed in the nuclei (Fig. 5C).

### Involvement of PCC1 in Light-Regulated Development

**Figure 6:**
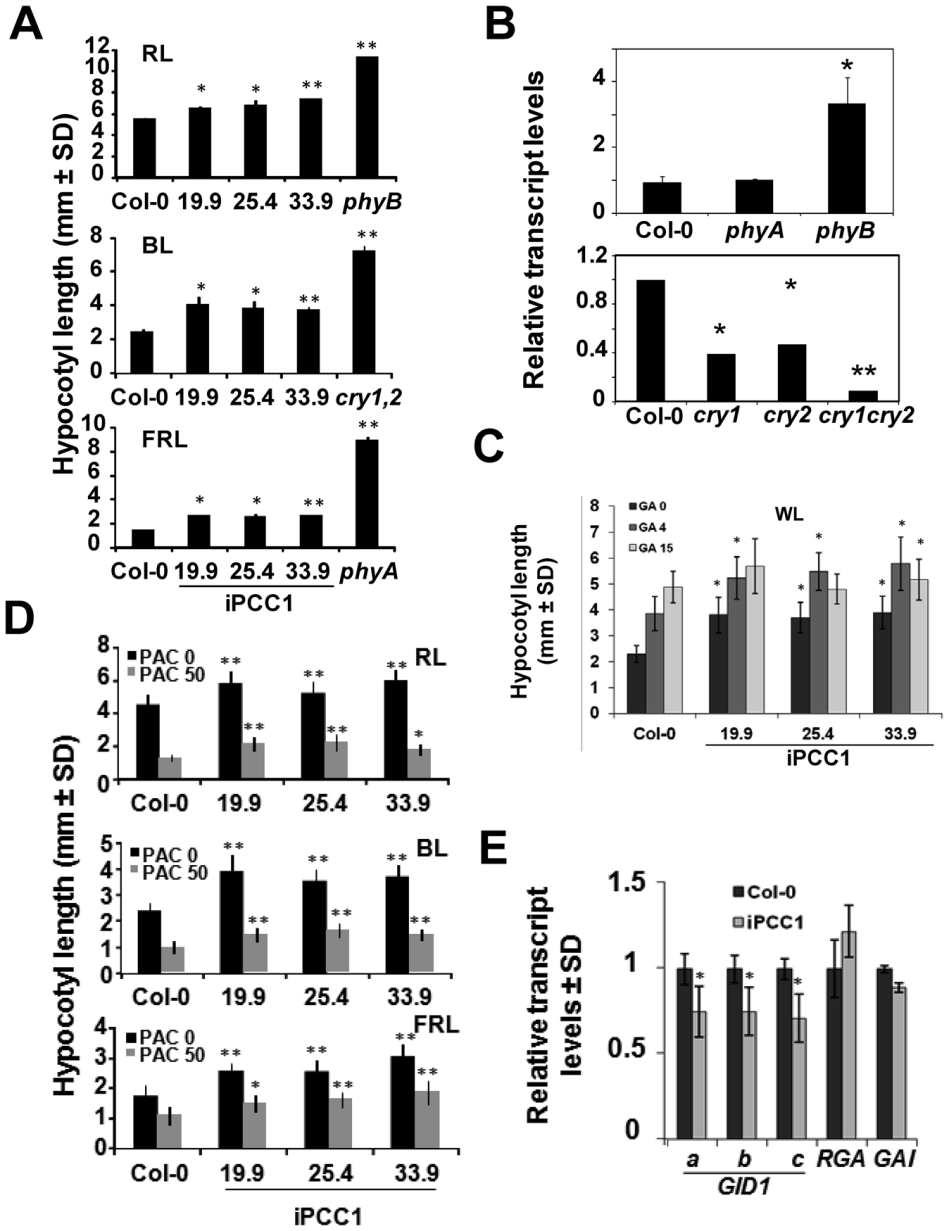
Light- and gibberellin-related hypocotyl phenotypes of iPCC1 plants. Hypocotyl length of seedlings grown under (A) 10 µmole m^−2^ s^−1^ of red light (RL), 30 µmole m^−2^ s^−1^ of blue light (BL), or 5 µmole m^−2^ s^−1^ of far-red light (FRL) for 4 days, and (D) the same light conditions as in (A) but treated as indicated with the gibberellin synthesis inhibitor paclobutrazol (PAC). (B) *PCC1* transcript levels were quantified by qRT-PCR in wild type Col-0 and the indicated photoreceptor mutant seedlings. Values are the mean of three independent biological replicates ± SD and are expressed as relative levels to those detected in wild type seedlings. (C) Hypocotyl length of seedlings grown under 60 µmole m^−2^ s^−1^ of white light (WL), with the indicated µM concentrations of GA3 (GA). Values are the mean of 20 hypocotyls per genotype and condition ± SD. * and ** represents statistically significant (p<0.05 or p<0.01, respectively, in Students t-test) different values in iPCC1 seedlings when compared to wild type seedlings under the same condition. (E) Transcript levels of the indicated genes involved in the perception and signaling of gibberellins were analyzed by qRT-PCR in Col-0 and iPCC1 seedlings grown under 16 h light/8 h darkness photoperiodic white light conditions for 14 days. Values are the mean of three independent biological replicates ± SD and are expressed as relative levels to those detected in wild type seedlings. * represents statistically significant (p<0.05 in Students t-test) different values in iPCC1 seedlings when compared to wild type seedlings.

PCC1 regulates flowering time, as demonstrated by the late flowering phenotype observed in iPCC1 plants grown under long day photoperiodic conditions [2]. However, this seems to be not related to interference with the function of the well characterized regulators of the photoperiod-dependent flowering pathway as demonstrated above. Alternatively, PCC1 control of this developmental transition might be related to other key factor such as light perception operating in this pathway. Next, we checked whether iPCC1 plants might display other light-related phenotypes. The photomorphogenic inhibition of hypocotyl elongation, cotyledon opening and acquisition of photosynthetic competence is controlled through light perception and downstream signaling involving Phytochrome Interacting Factors (PIFs) as well as gibberellin-related DELLA proteins [7], [8]. We analyzed whether iPCC1 seedlings displayed differential hypocotyl elongation phenotypes compared to wild type seedlings under different qualities of light. Figure 6A shows that iPCC1 hypocotyls grew longer than those of wild type seedlings under blue, red and far-red lights. Whereas iPCC1 hypocotyls were also longer than wild type ones under white light (Fig. 6C and Fig. S4), iPCC1 hypocotyls were not significantly longer than wild type under darkness (Fig. S4). Longer hypocotyls in far red, red, or blue lights might be related to deficiency in the perception of those qualities of lights through phytochromes and cryptochromes, respectively. However, based on comparative transcriptome analysis of iPCC1 vs wild type seedlings [33], no statistically significant change in the transcript levels of the phytochrome and cryptochrome encoding genes was detected (Table S1). Because iPCC1 hypocotyls were not as long as those from *phyA* mutant under far red light, *phyB* mutant under red light or as those from *cry1cry2* double mutant under blue light, we believe that iPCC1 seedlings are somehow partially blind to light in general, or possibly defective in downstream components of light signaling cascades that are common to different light qualities. Regarding this, the levels of *PCC1* transcript are up-regulated in the *phyB* mutant and, in turn, down-regulated in *cry1*, *cry2* and *cry1cry2* mutants when compared to wild type plants (Fig. 6B) suggesting that red and blue light signaling exerts negative and positive modulation, respectively, on *PCC1* gene expression. Since GAs promote hypocotyl elongation, we tested whether iPCC1 and wild type seedlings responded similarly to exogenous GAs under white light. Figure 6C shows that iPCC1 hypocotyls were as responsive as wild types to 4 µM GA_3_ treatment. By contrast, at 15 µM GA_3_ wild type hypocotyls were still responsive to GAs but iPCC1 hypocotyls were not responsive anymore (Fig. 6C). We also checked the effect of the GA synthesis inhibitor paclobutrazol (PAC) on hypocotyl elongation under different light qualities. The strong hypocotyl shortening effect exerted by PAC was observed in both wild type and iPCC1 hypocotyls grown under blue, red and far-red lights, but iPCC1 hypocotyls were still significantly longer than wild types in every condition tested (Fig. 6D). Because GA signaling is a key regulatory factor in the control of hypocotyl elongation, we tested by qRT-PCR the transcript levels of GA receptor and DELLA encoding genes in wild type and iPCC1 seedlings. Figure 6E shows that genes coding for the GA receptors, *GID1a*, *GID1b* and *GID1c*, were all slightly but significantly down-regulated in iPCC1 seedlings, thus supporting that GA perception might be altered in iPCC1 plants. Neither *RGA* nor *GAI* gene transcripts were significantly altered in iPCC1 seedlings (Fig. 6E). Whether deficient gibberellins perception might be responsible for the delayed flowering observed in iPCC1 plants will require further work. In summary, the partial skotomorphogenic phenotype under different light qualities and the altered sensitivity to GAs in iPCC1 plants as well as the opposite regulation exerted by PHYB and CRY photoreceptors on *PCC1* expression, point to PCC1 as an important regulatory node in photomorphogenic responses.

### Y2H-based Screening Reveals the Interaction between PCC1 and the Subunit 5 of the COP9 Signalosome

**Figure 7:**
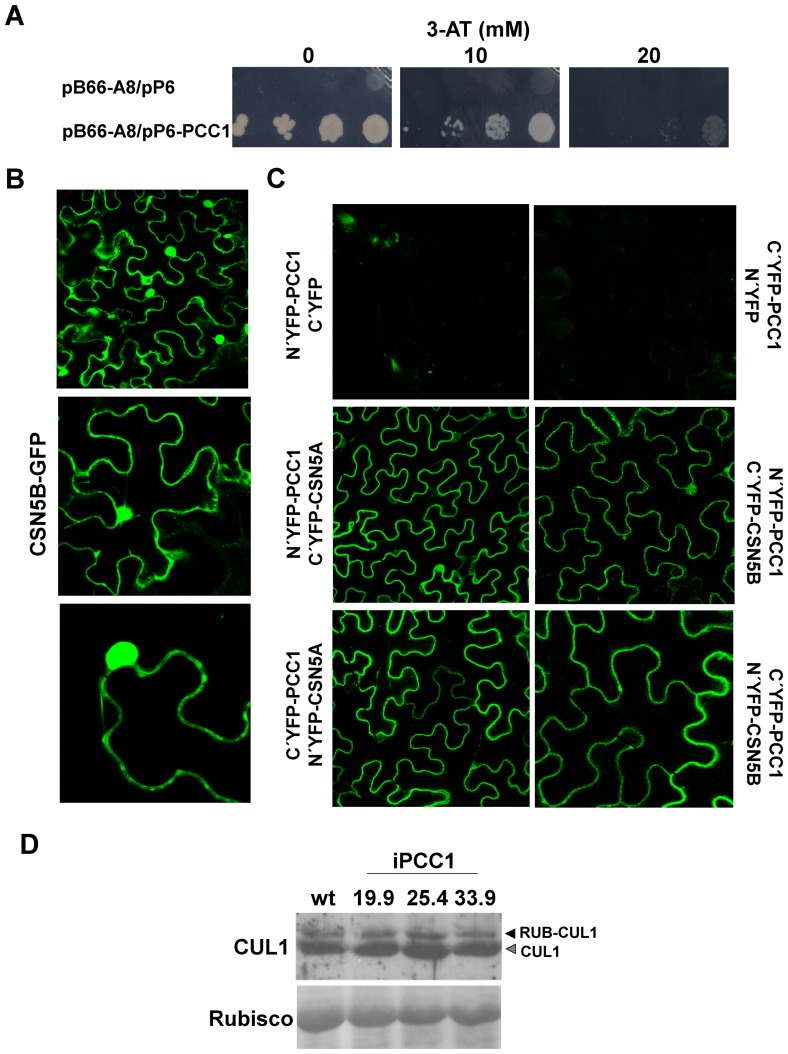
Functional interaction between PCC1 and the subunit 5 of the COP9 signalosome. (A) Growth in -Leu-Trp-His media of yeasts co-transformed with clone A8 corresponding to CSN5B subunit of COP9 signalosome fused to the *GAL4* activation domain (pB66-A8) and either PCC1 fused to the *GAL4* DNA binding domain (pP6-PCC1) or the empty vector (pP6). Growth was tested in increasing concentration of 3-aminotriazol (3-AT). (B) Subcellular localization of CSN5B in cytoplasm and nucleus of *Nicotiana benthamiana* leaves transformed with *35S::GFP-CSN5B*. (C) Interaction of PCC1 with CSN5A and CSN5B in the plasma membrane as demonstrated by BiFC in *Nicotiana benthamiana* leaves transiently co-transformed with the indicated constructs. (D) Levels of free and rubylated (RUB-) forms of CUL 1 in wt and iPCC1 plants as shown by Western blot with anti-CUL1 polyclonal antibodies. Ponceau S-stained Rubisco is shown as loading control.

To clarify the way PCC1 may exert regulation on light-regulated development we conducted a Y2H screening for protein interactors of PCC1. For that purpose, a truncated version of PCC1 containing the first 59 amino acids, thus lacking the C-terminal domain required for membrane anchoring, was fused to the DNA binding domain of *GAL4* (N-Gal4-PCC1(1-59)-C) and used as bait to screen an Arabidopsis library of cDNA clones from 7-day old Arabidopsis seedlings fused to the activation domain of *GAL4*. Among 31 positive clones detected out of 129 million possible interactions tested in the Y2H screening, 7 clones resulted to be either out of frame or antisense sequence clones and were consequently discarded. Another 21 positive clones corresponded to different sequences spanning the locus coding for the CSN5A subunit of the COP9 signalosome (CSN). It has been previously reported that CSN5A binds the DNA binding domain of *GAL4* and is thus frequently considered as a false positive interactor in many different Y2H screenings [34]. The rest 3 identical clones corresponded to the sequence between nucleotides 90 to 1207 of the At1g71230 coding for CSN5B/AJH2, the other subunit 5 of CSN. To confirm the strength of the interaction between PCC1 and CSN5B/AJH2, the clones isolated in the screening were co-transformed in *Saccharomyces cerevisiae* strain AH109 and the transformed yeast was plated in SC minimum medium (-His-Leu-Trp) supplemented with increasing concentrations of 3-aminotriazole (3-AT). Even at 20 mM 3-AT the growth of doubly transformed yeasts was clearly superior relative to the yeasts transformed with the GAL4AD-CSN5B and the empty GAL4BD- plasmids (Fig. 7A), thus suggesting a strong interaction between PCC1 and CSN5B. Because the Y2H system is an heterologous procedure to test plant protein-protein interactions caution is required when interpreting those data. The *in planta* interaction between two proteins must fulfill topological criteria with subcellular localizations being consistent with potential interaction. It has been reported that CSN5A in its monomeric form is located in the cytoplasm and nuclei [35]. We have transiently transformed *Nicotiana benthamina* with a *35S::GFP-CSN5B* construct and found that, similarly to CSN5A, fluorescence was detected in the cytoplasm and nucleus and it can not be ruled out the possibility that is also localized in the plasma membrane (Fig. 7B). By using BiFC, the interaction between PCC1 and both subunits CSN5A and CSN5B of CSN has been confirmed in Nicotiana. Figure 7C shows that the YFP fluorescence was reconstructed in the plasma membrane.

It has been widely characterized that CSN functions by cleaving the ubiquitin-like protein RUB/NEDD8 from cullins [36], which are essential components in the SCF (Skip1-cullin-F-box) complexes involved as E3 ubiquitin ligases in the ubiquitin-proteasome pathway, which is the preponderant protein turnover system in plants [37], [38]. RUB modification of cullins has been characterized to activate the E3 ubiquitin ligase activity [39] and thus CSN should function as a negative regulator of SCF complexes. However, genetic approaches have established that CSN functions *in vivo* promoting E3 ubiquitin ligase activity as a consequence of the protective effect exerted on SCF protein adaptors by limiting their autocatalytic degradation [40]. We have checked whether iPCC1 plants display altered CSN-mediated cleavage of cullin1 (CUL1) when compared to wild type plants. Figure 7D shows that iPCC1 displayed levels of RUB-CUL1 and CUL1 similar to wild type plants, which suggests that PCC1 does not interfere with de-rubylating activity of CSN, at least regarding to CUL1.

## Discussion

Despite its small size and the lack of well characterized domains informing about its potential functions, PCC1 protein has a significant impact on a wide array of physiological processes including polar lipid content, ABA-related responses and pathogen defense in Arabidopsis [33]. *PCC1* was initially identified as a gene with a circadian controlled pattern of expression and functionally related to defense against biotrophic pathogens [1]. It was further characterized as a gene with a strictly SA-dependent expression that seems to be involved in controlling both stress-induced and non stressed flowering [2]. Whereas the phenotypic effects of PCC1 gain- and loss-of function have been widely described [1], [2], [33], much less is known regarding the biochemistry and cell biology of PCC1 protein. Here we have characterized PCC1 as a plasma membrane associated protein that homodimerizes and requires its C-terminal domain to be anchored to the membrane. The C-terminus of PCC1 protein is a cysteine-rich domain that has being used to classify this protein as a member of the so-called Cysteine-rich Trans Membrane (CYSTM) domain-containing proteins [3]. This family of proteins has been proposed to be involved in resistance to stress and particularly against deleterious substances likely through the altered redox potential of the membrane due to the peculiar arrangement of several sulfhydryl groups within the membrane [3]. The alteration of the membrane redox potential might be determinant for quenching radical species or to affect the uptake of metal ions, which has been already reported for CDT1, another plant member of the CYSTM superfamily, involved in the exclusion of heavy metals in *Digitaria ciliaris* and *Oryza sativa* [41]. Besides, it has been also proposed that the N-terminal polar disordered head of the CYSTM properties might under certain conditions assume a certain degree of structure that could be important for alternative functions in the cytoplasm. In this regard, the disordered region upstream of the CYSTM domain has an amino acid composition and organization similar to prion-like proteins that may assume alternative conformations [3]. Although a truncated version of PCC1 lacking its CYSTM-containing C-terminal domain lost the specific plasma membrane localization of the full-length protein, we do not have data supporting whether CYSTM domain might be important for PCC1 function. Further comparative work with plants expressing either the full-length or truncated versions of PCC1 will help to clarify whether the different domains of PCC1 might be important for the regulatory roles exerted by this protein in previously described phenotypes or even in still uncharacterized new processes related to PCC1 function.

Our analysis of the spatial and temporal pattern of *PCC1* expression by using transgenic Arabidopsis plants expressing the *GUS* reporter gene under the control of a 1.1 kb promoter sequence of *PCC1* locus allowed to uncover a dual pattern of expression distinguishable both spatially and temporally. During the first days after seed germination, *PCC1* is expressed in the shoots only in the stomata guard cells and vascular tissues of cotyledons. After that, the transition from vegetative to reproductive shoot apical meristem correlates with the lower *PCC1* expression detected in stomata along with its higher expression in the vascular tissue connecting the apical meristem with the leaves through the vasculature in petioles and basal part of the leaves. Thereafter, *PCC1* expression is expanded all over the leaves. This dual pattern of *PCC1* expression is likely associated to different functions exerted by PCC1. The stomata-associated *PCC1* expression at early stages of development may be related to defense. In turn, coincidentally with the transition from vegetative to reproductive shoot apical meristem, signaling events originated in the SAM, and translocated to the leaves through the vasculature modulate *PCC1* expression for further developmental control of different responses. Because stomata has been widely characterized as entry sites for different kind of pathogens [42], the expression of *PCC1* in stomata guard cells at early post-germinative development might be connected to defense-related functions of PCC1. This hypothesis is consistent with previously reported phenotypes of enhanced resistance of plants overexpressing *PCC1* gene to the oomycete *Hyaloperonospora parasitica* [1] and also with enhanced susceptibility to *Phytophtora brassicae* and enhanced resistance to *Botrytis cinerea* observed for iPCC1 plants [33]. A potential defensive role of PCC1 in stomata might be especially important at early developmental stages of the plant, when physical barrier in cotyledons may not be completely formed. After cotyledons become fully expanded and developed, PCC1 might not be required in stomata for defense and its expression is turned-down in those specialized cells. A second distinguishable function for PCC1 emerges as plants get closer to the developmental transition from vegetative to reproductive shoot apical meristem. A vascular tissue-associated pattern of *PCC1* expression was visualized in *pPCC1::GUS* transgenic lines and it expanded from SAM to the basal part of leaves through leaf petioles. This spatial pattern of expression is interestingly coincident in time with the FT translocation from leaves to SAM [43], which is required to activate the expression of reproductive meristem identity genes such as *AP1* [44] and starting flowering primordia. Despite coincidences suggesting that PCC1 might be involved or interfere with the photoperiod-dependent flowering pathway [2], we have not found evidences demonstrating that PCC1 either interacts with the main players in the photoperiod-dependent flowering pathway, namely CO and FT, or interferes in *FT* activation by CO.

Our results suggest that the potential regulatory role exerted by PCC1 in flowering under inductive conditions should be connected with other factors involved in this developmental transition. We have presented experimental evidence supporting functional interactions between PCC1 and both light- and gibberellins-related signaling pathways (Fig. 6). The involvement of either light or gibberellins in regulating the transition to flowering is widely documented [45]. It has been recently reported that under long days, GA promotes the transition to flowering independently of CONSTANS (CO) and GIGANTEA (GI) by activating the expression of flowering time integrator genes such as FLOWERING LOCUS T (FT) and TWIN SISTER OF FT (TSF) in leaves [46]. This work provides a potential mechanistic explanation of PCC1 being involved in the perception and downstream signaling of stimuli such as light and gibberellins through functional interference with the CSN-mediated role in controlling the activity of multiple E3 ubiquitin ligases. By using BiFC, we have found that interaction between PCC1 and either CSN5A or CSN5B occurs in the plasma membrane (Figure 7). Although CSN5A and CSN5B are localized in cytoplasm and nucleus [35] (Figure 7B) the possibility that they are also partially localized in the plasma membrane cannot be ruled out. This potential plasma membrane localization of CSN5B would allow the interaction with PCC1. However, even if CSN5 subunits are not localized in the plasma membrane in wild type plants, it might be recruited to the membrane upon over-expression of PCC1. In this latter case, the fluorescence that is reconstructed in the plasma membrane in BiFC experiments would correspond to only a fraction of the total CSN5 protein present in the cell. Regarding this, the fact that the yeast 2-hybrid approach we used to identify the interaction between CSN5 and PCC1 was based on the use of a truncated version of PCC1 lacking its C-terminus as bait, suggest that the interaction occurs through the N-terminal domain of PCC1, which is oriented towards the cytoplasm. The interaction of the N-terminus of PCC1 with the cytoplasmic fraction of CSN5 would lead to the recruitment of at least part of the CSN5 subunits to the plasma membrane.

Altered functional interaction between PCC1 and CSN might subsequently alter the ubiquitination status of multiple proteins involved in sensing and transducing multiple stimuli. We have observed that PCC1 interacts with CSN5A and CSN5B subunits, recruiting them to the plasma membrane. Two different scenarios might be considered in the functional interaction between PCC1 and CSN5. This process might scavenge CSN5 subunits preventing the formation of CSN complex and eventually its nuclear translocation. Such a mechanism would imply that iPCC1 plants should have a reduced CSN function in cleaving RUB protein from modified cullins, and such significant alterations have not been detected at least for CUL1 in iPCC1 plants relative to wild type plants. It might be also possible that PCC1-mediated scavenging of CSN5 in the plasma membrane might avoid the assembly of a full CSN complex to be further translocated to the nucleus and function as a negative modulator of COP1-mediated skotomorphogenesis. Accordingly, iPCC1 plants lacking this scavenging mechanism showed partial skotomorphogenic phenotypes under different light conditions (Fig. 6A). However, CSN complex not only modulates COP1 but many other E3 ubiquitin ligases. A model like that would explain that defective CSN function would lead RUB-cullin to accumulate and to keep E3 ubiquitin ligases in their active form. Although we have not observed alterations in CUL1 rubylation we can not rule out the possibility that other cullins may be affected. Alternatively, the function of PCC1 interacting with CSN5 at the plasma membrane might be a mechanism of recruitment of CSN5 to some membrane-associated target acting as potential substrate for CSN5-mediated hydrolytic activity. Whether PCC1-CSN5 interaction could be part of a general regulatory mechanism in regulating the ubiquitination status of multiple targets or a more specific membrane-associated mechanism affecting particular F-box proteins involved in light- and gibberellin-regulated processes, such as photomorphogenesis or flowering time, or others involved in defense against varied pathogens will require further work.

Despite multiple mechanistic explanations of PCC1 function as regulator of the transition to flowering and photomorphogenesis remain to be clarified, a simple model consistent with the potential functional interaction and the observed phenotypic effects can be drawn. We have previously reported that PCC1 is a positive regulator of the transition to flowering [2]. On the other hand, PHYB seems to promote CO degradation and thus act as a negative regulator of flowering time, whereas, CRY1 and CRY2 stimulate CO accumulation under blue light, and they are thus positive regulators of the transition to flowering [47]–[49]. These data suggest that a model where PHYB and CRY1,2 regulate *PCC1* expression negatively and positively, respectively, is consistent with the flowering time phenotype of photoreceptor mutants and iPCC1 loss-of-function plants. Moreover, the fact that no altered transcript levels of the genes encoding phytochromes and cryptochromes was observed when compared iPCC1 versus wild type plants suggests that PCC1 is not controlling light perception through direct effects on the genes coding for photoreceptors. Alternatively, PCC1 could exert an effect on the inputs of the circadian clock or directly on the function of the oscillator itself. Remarkably, the endogenous content of salicylic acid (SA) levels, which is a potent activator of PCC1 expression, seems to regulate the expression of genes coding for components of the circadian clock such as *CCA1* [2]. An increase in the *CCA1* transcript levels and a concomitant reduction in *CO* transcript were detected at dawn in SA-deficient plants, defective in *PCC1* expression, when compared with Col-0 plants [2]. We believe that the potential regulatory effect exerted by PCC1 on components of the circadian oscillator would be more important for its role in regulating flowering time than the effect on CO. A still unknown output of the circadian clock would be a potential target for the oscillator-mediated effect triggered by PCC1 in controlling flowering time. Clearly, more work is required to elucidate the mechanism underlying PCC1 control on flowering.

Regarding the photomorphogenesis phenotype, phytochromes and cryptochromes as well as PCC1 are positive regulators of photomorphogenesis and the corresponding mutants are thus skototmorphogenic. However, iPCC1 plants display only partial skotomorphogenic phenotype thus suggesting PCC1 may be involved in light signal transduction downstream photoreceptors together with other functionally-related proteins. CSN accumulates in the nuclei under darkness then promoting the COP1-mediated degradation of positive regulators of photomorphogenesis, such as HY5, and thus promoting skotomorphogenesis. However, under light CSN accumulates in the cytoplasm thus releasing HY5 from degradation and allowing photomoprphogenesis. In this context, PCC1-CSN5 interaction in the plasma membrane would function as a scavenging system to prevent cytoplasm to nucleus CSN translocation and then helping photomorphogenic conditions. Accordingly, plants with defective PCC1 function would lack this scavenging activity thus allowing CSN to be localized in the nuclei promoting skotomorphogenesis. However, because PHYB is a negative regulator of *PCC1* expression as described in this work, we believe that PCC1 positive effect on photomorphogenesis should be more related to CRY1,2 function. Regarding this, PCC1 might somehow regulate the interaction between CRY1 and SUPRESSOR OF PHYA1 (SPA1), which under blue light allows the inactivation of COP1 with the consequent promotion of photomorphogenesis [50], [51].

## Supporting Information

Figure S1 **Co-localization of GFP-PCC1 with stained membranes.** Membranes were stained with fluorescent lipid stain FM64 in *Nicotiana benthamiana* leaves transformed with *35S::GFP-PCC1* and *35S::GFP-Δ177-PCC1* constructs expressing the full and truncated versions of tagged PCC1 proteins. Overlays of green and red fluorescence due to GFP and FM64 are shown in the right panels and yellow appeared only when co-localization occurred.
(PDF)

Figure S2 **PCC1-GFP is localized only in the plasma membrane in plasmolysed cells of transformed Nicotiana leaves.** *Nicotina benthamiana* leaves transiently transformed with *35S::PCC1-GFP* construct were either infiltrated with water (control) or with 0.5 M sorbitol (plasmolysed) and 6 h after infiltration fluorescence was observed under confocal microscopy. Cell contour is marked with red dashed lines.
(PDF)

Figure S3 **PCC1 is not localized in plastids.** Green fluorescence due to GFP and red fluorescence due to FM64 in membranes and to chlorophyll in plastids (pointed by arrows) are shown together with the corresponding bright field photopgraphs.
(PDF)

Figure S4 **Hypocotyl length of Col-0 wild type and iPCC1 seedlings grown under white light or darkness.** Values are the mean of 20 hypocotyls per genotype ± SD. * and ** represents statistically significant (p<0.05 or p<0.01, respectively, in Students t-test) different values in iPCC1 or phyB seedlings when compared to wild type seedlings under the same condition. No statistically significant differences were observed following Students t-test for the seedlings grown under darkness.
(PDF)

Table S1 Comparative transcript levels of phytochrome (PHY) and cryptochrome (CRY) encoding genes in iPCC1 vs Col-0 seedlings.
(PDF)

## References

[1] Sauerbrunn N, Schlaich NL (2004) PCC1: a merging point for pathogen defence and circadian signalling in Arabidopsis. Planta 218: 552–561. 14614626 10.1007/s00425-003-1143-z

[2] Segarra S, Mir R, Martínez C, León J (2010) Genome-wide analyses of the transcriptomes of salicylic acid-deficient versus wild type plants uncover Pathogen and Circadian Controlled 1 (PCC1) as a regulator of flowering time in Arabidopsis. Plant Cell Environ 33: 11–22. 19781011 10.1111/j.1365-3040.2009.02045.x

[3] Venancio TM, Aravind L (2010) CYSTM a novel cysteine-rich transmembrane module with a role in stress tolerance across eukaryotes. Bioinformatics 26: 149–152. 19933165 10.1093/bioinformatics/btp647 PMC2804304

[4] Lau OS, Deng XW (2010) Plant hormone signaling lightens up: integrators of light and hormones. Curr Opin Plant Biol 13: 571–517. 20739215 10.1016/j.pbi.2010.07.001

[5] Schwechheimer C (2011) Gibberellin signaling in plants - the extended version. Front. Plant Sci 2: 107. 10.3389/fpls.2011.00107 PMC3355746 22645560

[6] Seo M, Nambara E, Choi G, Yamaguchi S (2009) Interaction of light and hormone signals in germinating seeds. Plant Mol Biol 69: 463–472. 19031046 10.1007/s11103-008-9429-y

[7] De Lucas M, Daviére JM, Rodrigues-Falcon M, Iglesias-Pedraz JM, Lorrain S, et al (2008) A molecular framework for light and gibberellin control of cell elongation. Nature 451: 480–484. 18216857 10.1038/nature06520

[8] Feng S, Martínez C, Gusmaroli G, Wang Y, Zhou J, et al (2008) Coordinated regulation of Arabidopsis thaliana development by light and gibberellins. Nature 451: 475–479. 18216856 10.1038/nature06448 PMC2562044

[9] Mutasa-Göttgens E, Hedden P (2009) Gibberellin as a factor in floral regulatory networks. J Exp Bot 60: 1979–1989. 19264752 10.1093/jxb/erp040

[10] Bastian R, Dawe A, Meier S, Ludidi N, Bajic VB, et al (2010) Gibberellic acid and cGMP-dependent transcriptional regulation in Arabidopsis thaliana. Plant Signal Behav 5: 224–232. 20118660 10.4161/psb.5.3.10718 PMC2881265

[11] Yu S, Galvão VC, Zhang YC, Horrer D, Zhang TQ, et al (2012) Gibberellin regulates the Arabidopsis floral transition through miR156-targeted SQUAMOSA promoter binding-like transcription factors. Plant Cell 24: 3320–3332. 22942378 10.1105/tpc.112.101014 PMC3462634

[12] Arc E, Galland M, Cueff G, Godin B, Lounifi I, et al (2011) Reboot the system thanks to protein post-translational modifications and proteome diversity: How quiescent seeds restart their metabolism to prepare seedling establishment. Proteomics 11: 1606–1618. 21433284 10.1002/pmic.201000641

[13] Dill A, Thomas SG, Hu J, Steber CM, Sun TP (2004) The Arabidopsis F-box protein SLEEPY1 targets gibberellin signaling repressors for gibberellin-induced degradation. Plant Cell 16: 1392–1405. 15155881 10.1105/tpc.020958 PMC490034

[14] Wang F, Deng XW (2011) Plant ubiquitin-proteasome pathway and its role in gibberellin signaling. Cell Res 21: 1286–1294. 21788985 10.1038/cr.2011.118 PMC3193469

[15] Hotton SK, Callis J (2008) Regulation of cullin RING ligases. Annu Rev Plant Biol 59: 467–489. 18444905 10.1146/annurev.arplant.58.032806.104011

[16] Cope GA, Suh GS, Aravind L, Schwarz SE, Zipursky SL, et al (2002) Role of predicted metalloprotease motif of Jab1/Csn5 in cleavage of Nedd8 from Cul1. Science 298: 608–611. 12183637 10.1126/science.1075901

[17] Gusmaroli G, Figueroa P, Serino G, Deng XW (2007) Role of the MPN subunits in COP9 signalosome assembly and activity, and their regulatory interaction with Arabidopsis Cullin3-based E3 ligases. Plant Cell 19: 564–581. 17307927 10.1105/tpc.106.047571 PMC1867349

[18] Serino G, Deng XW (2003) The COP9 signalosome: regulating plant development through the control of proteolysis. Annu Rev Plant Biol 54: 165–182. 14502989 10.1146/annurev.arplant.54.031902.134847

[19] Stratmann JW, Gusmaroli G (2012) Many jobs for one good cop - the COP9 signalosome guards development and defense. Plant Sci 185–186: 50–64. 10.1016/j.plantsci.2011.10.004 22325866

[20] Lozano-Juste J, León J (2011) Nitric oxide regulates DELLA content and PIF expression to promote photomorphogenesis in Arabidopsis. Plant Physiol 156: 1410–1423. 21562334 10.1104/pp.111.177741 PMC3135954

[21] Nakagawa T, Kurose T, Hino T, Tanaka K, Kawamukai M, et al (2007) Development of series of gateway binary vectors, pGWBs, for realizing efficient construction of fusion genes for plant transformation. J Biosci Bioeng 104: 34–41. 17697981 10.1263/jbb.104.34

[22] Fromont-Racine M, Rain JC, Legrain P (1997) Toward a functional analysis of the yeast genome through exhaustive two-hybrid screens. Nat Genet 16: 277–282. 9207794 10.1038/ng0797-277

[23] Belda-Palazón B, Ruiz L, Martí E, Tárraga S, Tiburcio AF, et al.. (2012) Aminopropyltransferases involved in polyamine biosynthesis localize preferentially in the nucleus of plant cells. PLoS One 7(10), e46907. 10.1371/journal.pone.0046907 PMC3466176 23056524

[24] Simon R, Igeño MI, Coupland G (1996) Activation of floral meristem identity genes in Arabidopsis. Nature 384: 59–62. 8900276 10.1038/384059a0

[25] Martínez C, Pons E, Prats G, León J (2004) Salicylic acid regulates flowering time and links defence responses and reproductive development. Plant J 37: 209–217. 14690505 10.1046/j.1365-313x.2003.01954.x

[26] Kyte J, Doolittle RF (1982) A simple method for displaying the hydropathic character of a protein. J Mol Biol 157: 105–132. 7108955 10.1016/0022-2836(82)90515-0

[27] Marmagne A, Rouet MA, Ferro M, Rolland N, Alcon C, et al (2004) Identification of new intrinsic proteins in Arabidopsis plasma membrane proteome. Mol Cell Proteomics 3: 675–691. 15060130 10.1074/mcp.M400001-MCP200

[28] Nühse TS, Stensballe A, Jensen ON, Peck SC (2004) Phosphoproteomics of the Arabidopsis plasma membrane and a new phosphorylation site database. Plant Cell 16: 2394–2405. 15308754 10.1105/tpc.104.023150 PMC520941

[29] Hofmann K, Stoffel W (1993) TMbase - A database of membrane spanning proteins segments. Biol Chem Hoppe-Seyler 374: 166.

[30] Kobayashi Y, Weigel D (2007) Move on up, it's time for change--mobile signals controlling photoperiod-dependent flowering. Genes Dev 21: 2371–2384. 17908925 10.1101/gad.1589007

[31] Jaeger KE, Wigge PA (2007) FT protein acts as a long-range signal in Arabidopsis. Curr Biol 17: 1050–1054. 17540569 10.1016/j.cub.2007.05.008

[32] Mathieu J, Warthmann N, Küttner F, Schmid M (2007) Export of FT protein from phloem companion cells is sufficient for floral induction in Arabidopsis. Curr Biol 17: 1055–1060. 17540570 10.1016/j.cub.2007.05.009

[33] Mir R, Hernández ML, Abou-Mansour E, Martínez-Rivas JM, Mauch F, et al (2013) Pathogen and Circadian Controlled 1 (PCC1) regulates polar lipid content, ABA-related responses, and pathogen defence in *Arabidopsis thaliana* . J Exp Bot 64: 3385–3395. 23833195 10.1093/jxb/ert177

[34] Nordgård O, Dahle Ø, Andersen TØ, Gabrielsen OS (2001) JAB1/CSN5 interacts with the GAL4 DNA binding domain: a note of caution about two-hybrid interactions. Biochimie 83: 969–971. 11728635 10.1016/s0300-9084(01)01329-3

[35] Kwok SF, Staub JM, Deng XW (1999) Characterization of two subunits of Arabidopsis 19S proteasome regulatory complex and its possible interaction with the COP9 complex. J Mol Biol 285: 85–95. 9878390 10.1006/jmbi.1998.2315

[36] Nezames CD, Deng XW (2012) The COP9 signalosome: its regulation of cullin-based E3 ubiquitin ligases and role in photomorphogenesis. Plant Physiol 160: 38–46. 22715109 10.1104/pp.112.198879 PMC3440213

[37] Moon J, Parry G, Estelle M (2004) The ubiquitin-proteasome pathway and plant development. Plant Cell 16: 3181–3195. 15579807 10.1105/tpc.104.161220 PMC535867

[38] Dreher K, Callis J (2007) Ubiquitin, hormones and biotic stress in plants. Ann Bot 99: 787–822. 17220175 10.1093/aob/mcl255 PMC2802907

[39] Parry G, Estelle M (2004) Regulation of cullin-based ubiquitin ligases by the Nedd8/RUB ubiquitin-like proteins. Semin Cell Dev Biol 15: 221–229. 15209382 10.1016/j.semcdb.2003.12.003

[40] Wee S, Geyer RK, Toda T, Wolf DA (2005) CSN facilitates Cullin-RING ubiquitin ligase function by counteracting autocatalytic adapter instability. Nat Cell Biol 7: 387–391. 15793566 10.1038/ncb1241

[41] Kuramata M, Masuya S, Takahashi Y, Kitagawa E, Inoue C, et al (2009) Novel cysteine-rich peptides from Digitaria ciliaris and Oryza sativa enhance tolerance to cadmium by limiting its cellular accumulation. Plant Cell Physiol 50: 106–117. 19017626 10.1093/pcp/pcn175

[42] Zeng W, Melotto M, He SY (2010) Plant stomata: a checkpoint of host immunity and pathogen virulence. Curr Opin Biotechnol 21: 599–603. 20573499 10.1016/j.copbio.2010.05.006 PMC2946497

[43] Wigge PA (2011) FT, a mobile developmental signal in plants. Curr Biol 21: R374–378. 21549960 10.1016/j.cub.2011.03.038

[44] Kardailsky I, Shukla VK, Ahn JH, Dagenais N, Christensen SK, et al (1999) Activation tagging of the floral inducer FT. Science 286: 1962–1965. 10583961 10.1126/science.286.5446.1962

[45] Srikanth A, Schmid M (2011) Regulation of flowering time: all roads lead to Rome. Cell Mol Life Sci 68: 2013–2037. 21611891 10.1007/s00018-011-0673-y PMC11115107

[46] Galvão VC, Horrer D, Küttner F, Schmid M (2012) Spatial control of flowering by DELLA proteins in Arabidopsis thaliana. Development 139: 4072–4082. 22992955 10.1242/dev.080879

[47] Cerdán PD, Chory J (2003) Regulation of flowering time by light quality. Nature 423: 881–885. 12815435 10.1038/nature01636

[48] Guo H, Yang H, Mockler TC, Lin C (1998) Regulation of flowering time by Arabidopsis photoreceptors. Science 279: 1360–1363. 9478898 10.1126/science.279.5355.1360

[49] Mockler T, Guo H, Yang H, Duong H, Lin C (1999) Antagonistic actions of Arabidopsis cryptochromes and phytochrome B in the regulation of floral induction. Development 126: 2073–2082. 10207133 10.1242/dev.126.10.2073

[50] Liu B, Zuo Z, Liu H, Liu X, Lin C (2011) Arabidopsis cryptochrome 1 interacts with SPA1 to suppress COP1 activity in response to blue light. Genes Dev 25: 1029–1034. 21511871 10.1101/gad.2025011 PMC3093118

[51] Weidler G, Zur Oven-Krockhaus S, Heunemann M, Orth C, Schleifenbaum F, et al (2012) Degradation of Arabidopsis CRY2 is regulated by SPA proteins and phytochrome A. . Plant Cell 24: 2610–2623. 22739826 10.1105/tpc.112.098210 PMC3406922

