# Supplementary material for: Pathogen and Circadian Controlled 1 (PCC1) Protein Is Anchored to the Plasma Membrane and Interacts with Subunit 5 of COP9 Signalosome in Arabidopsis
Source: PLoS One. 2014 Jan 27;9(1):e87216. doi: 10.1371/journal.pone.0087216 (PMC3903633; doi:10.1371/journal.pone.0087216)
Supplement: Figure S1 — Co-localization of GFP-PCC1 with stained membranes. Membranes were stained with fluorescent lipid stain FM64 in Nicotiana benthamiana leaves transformed with 35S::GFP-PCC1 and 35S::GFP-Δ177-PCC1 constructs expressing the full and truncated versions of tagged PCC1 proteins. Overlays of green and red fluorescence due to GFP and FM64 are shown in the right panels and yellow appeared only when co-localization occurred. (PDF) [file pone.0087216.s001.pdf]

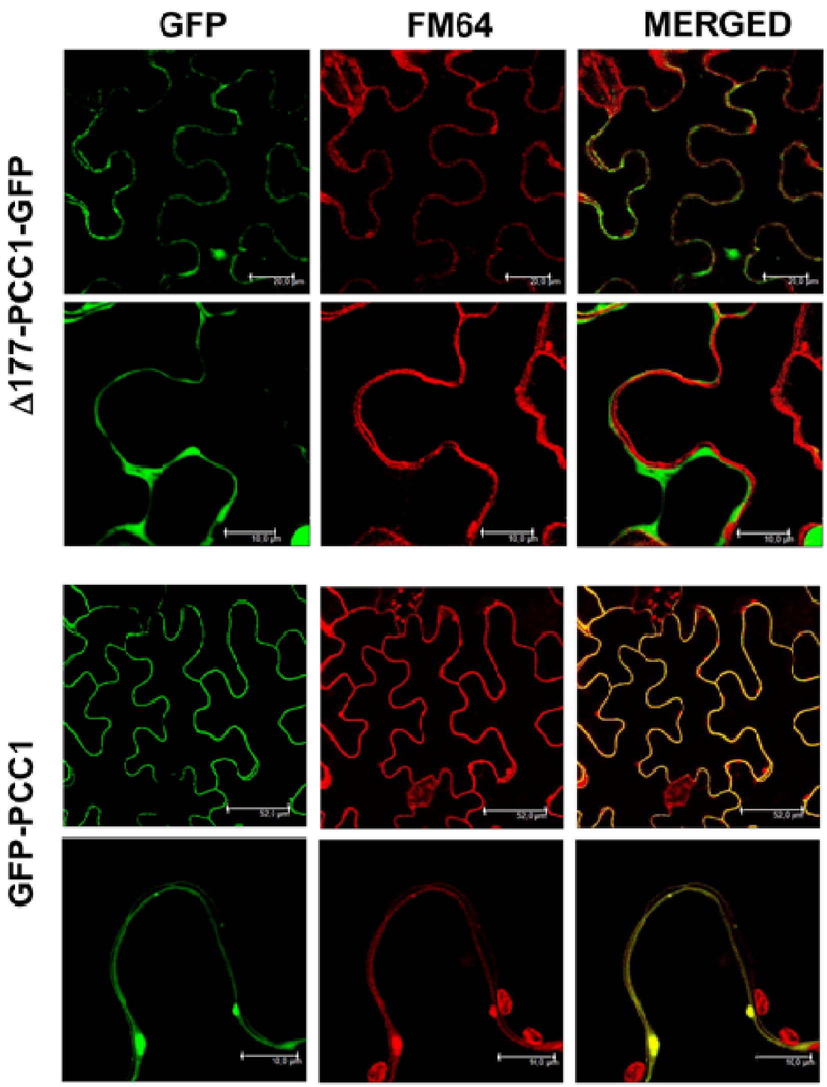

Figure S1 Co-localization of GFP-PCC1 with stained membranes. Membranes were stained with fluorescent lipid stain FM64 in *Nicotiana benthamiana* leaves transformed with *35S::GFP-PCC1* and *35S::GFP-Δ177-PCC1* constructs expressing the full and truncated versions of tagged PCC1 proteins. Overlays of green and red fluorescence due to GFP and FM64 are shown in the right panels and yellow appeared only when co-localization occurred.
