# Supplementary material for: Pathogen and Circadian Controlled 1 (PCC1) Protein Is Anchored to the Plasma Membrane and Interacts with Subunit 5 of COP9 Signalosome in Arabidopsis
Source: PLoS One. 2014 Jan 27;9(1):e87216. doi: 10.1371/journal.pone.0087216 (PMC3903633; doi:10.1371/journal.pone.0087216)
Supplement: Figure S2 — PCC1-GFP is localized only in the plasma membrane in plasmolysed cells of transformed Nicotiana leaves. Nicotina benthamiana leaves transiently transformed with 35S::PCC1-GFP construct were either infiltrated with water (control) or with 0.5 M sorbitol (plasmolysed) and 6 h after infiltration fluorescence was observed under confocal microscopy. Cell contour is marked with red dashed lines. (PDF) [file pone.0087216.s002.pdf]

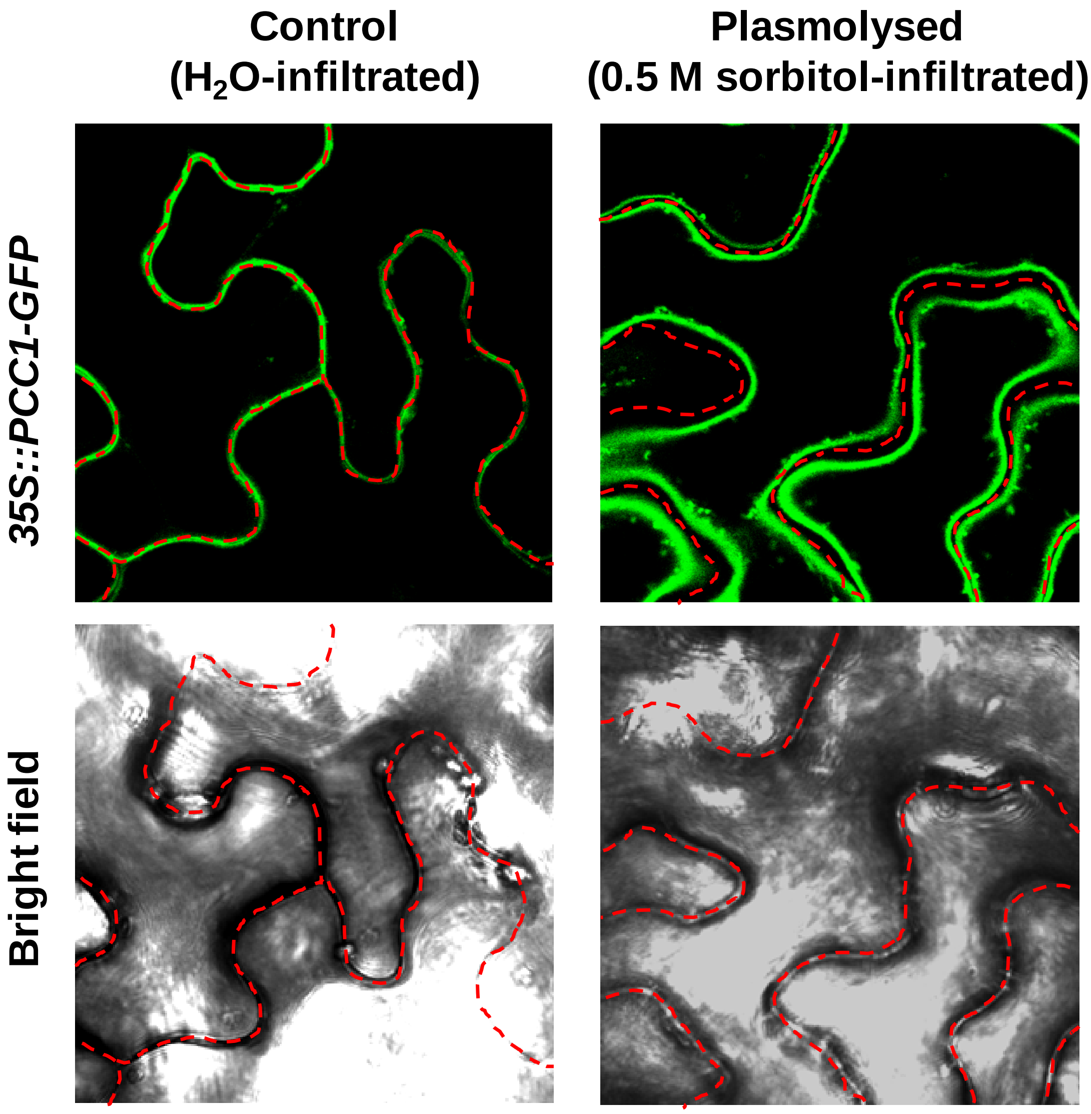

Figure S2 PCC1-GFP is localized only in the plasma membrane in plasmolysed cells of transformed Nicotiana leaves. *Nicotina benthamiana* leaves transiently transformed with *35S::PCC1-GFP* construct were either infiltrated with water (control) or with 0.5 M sorbitol (plasmolysed) and 6 h after infiltration fluorescence was observed under confocal microscopy. Cell perimeters are marked with red dashed lines.
