# Supplementary material for: Pathogen and Circadian Controlled 1 (PCC1) Protein Is Anchored to the Plasma Membrane and Interacts with Subunit 5 of COP9 Signalosome in Arabidopsis
Source: PLoS One. 2014 Jan 27;9(1):e87216. doi: 10.1371/journal.pone.0087216 (PMC3903633; doi:10.1371/journal.pone.0087216)
Supplement: Figure S3 — PCC1 is not localized in plastids. Green fluorescence due to GFP and red fluorescence due to FM64 in membranes and to chlorophyll in plastids (pointed by arrows) are shown together with the corresponding bright field photopgraphs. (PDF) [file pone.0087216.s003.pdf]

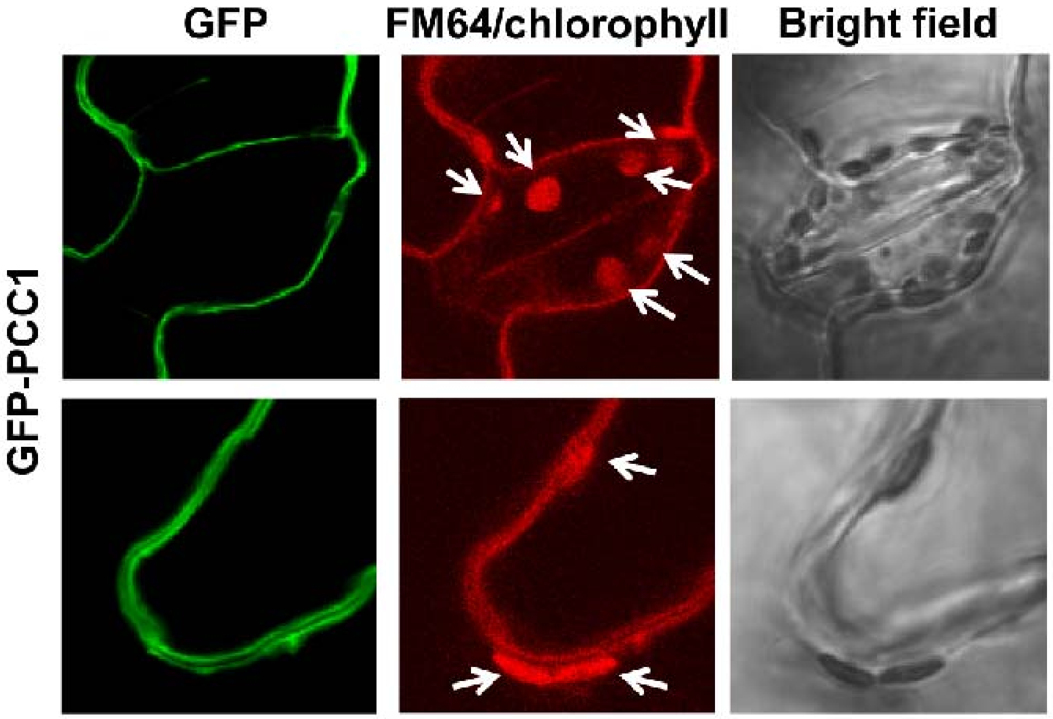

Figure S3 PCC1 is not localized in plastids. Green fluorescence due to GFP and red fluorescence due to FM64 in membranes and to chlorophyll in plastids (pointed by arrows) are shown together with the corresponding bright field photopgraphs.
