# Supplementary material for: Pathogen and Circadian Controlled 1 (PCC1) Protein Is Anchored to the Plasma Membrane and Interacts with Subunit 5 of COP9 Signalosome in Arabidopsis
Source: PLoS One. 2014 Jan 27;9(1):e87216. doi: 10.1371/journal.pone.0087216 (PMC3903633; doi:10.1371/journal.pone.0087216)
Supplement: Figure S4 — Hypocotyl length of Col-0 wild type and iPCC1 seedlings grown under white light or darkness. Values are the mean of 20 hypocotyls per genotype ± SD. * and ** represents statistically significant (p<0.05 or p<0.01, respectively, in Students t-test) different values in iPCC1 or phyB seedlings when compared to wild type seedlings under the same condition. No statistically significant differences were observed following Students t-test for the seedlings grown under darkness. (PDF) [file pone.0087216.s004.pdf]

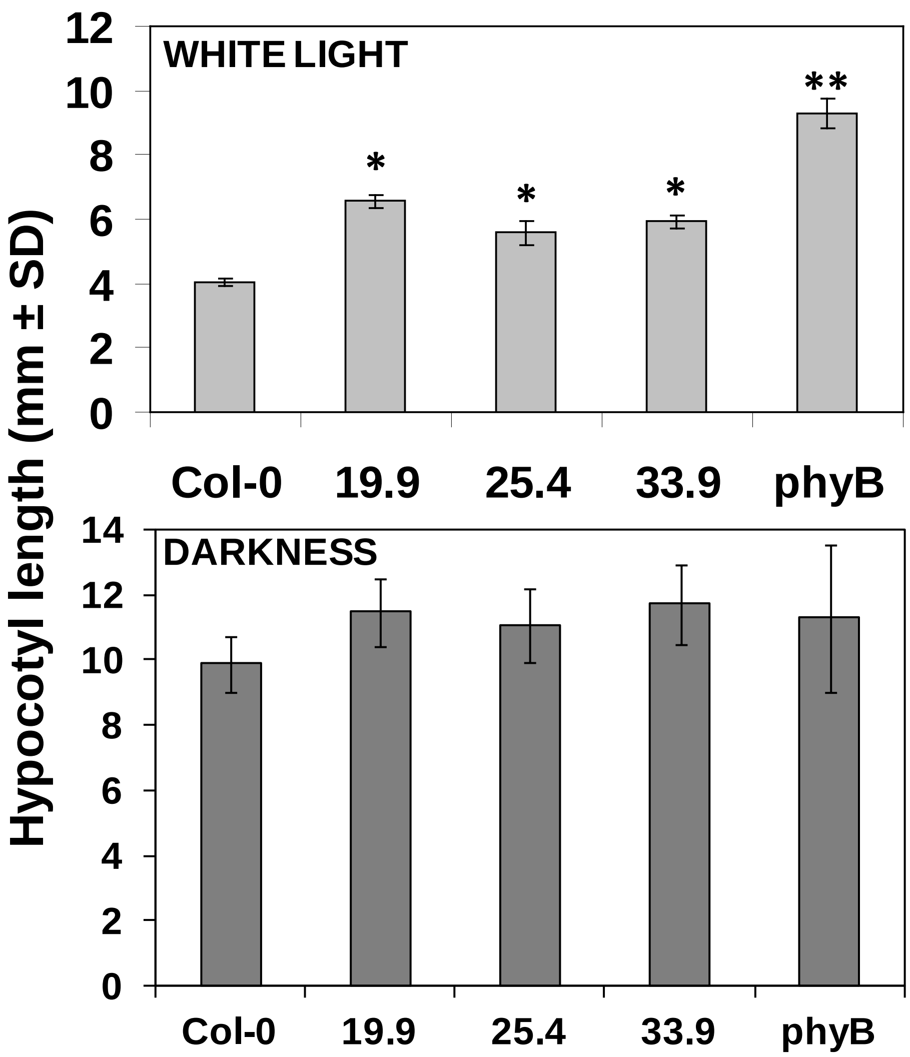

Figure S4 Hypocotyl length of Col-0 wild type and iPCC1 seedlings grown under white light or darkness. Values are the mean of 20 hypocotyls per genotype ± SD. * and ** represents statistically significant ($p < 0.05$ or $p < 0.01$, respectively, in Student´s t-test) different values in iPCC1 or phyB seedlings when compared to wild type seedlings under the same condition. No statistically significant differences were observed following Student´s t-test for the seedlings grown under darkness.
