# Supplementary material for: Pathogen and Circadian Controlled 1 (PCC1) Protein Is Anchored to the Plasma Membrane and Interacts with Subunit 5 of COP9 Signalosome in Arabidopsis
Source: PLoS One. 2014 Jan 27;9(1):e87216. doi: 10.1371/journal.pone.0087216 (PMC3903633; doi:10.1371/journal.pone.0087216)
Supplement: Table S1 — Comparative transcript levels of phytochrome (PHY) and cryptochrome (CRY) encoding genes in iPCC1 vs Col-0 seedlings. (PDF) [file pone.0087216.s005.pdf]

Table S1. Comparative transcript levels of phytochrome (PHY) and cryptochrome (CRY) encoding genes in iPCC1 vs Col-0 seedlings.

Fold Change	FDR (LiMMA)	Probe ID	Transcript ID	Annotation
1.23	0.2197	264508_at	At1g09570	PHYA
1.23	0.1082	266065_at	At2g18790	PHYB
-1.29	0.2613	249666_at	At5g35840	PHYC
-1.23	0.3450	245487_at	At4g16250	PHYD
1.04	0.7643	254680_at	At4g18130	PHYE
1.20	0.3895	255068_at	At4g08920	CRY1
1.07	0.7859	263669_at	At1g04400	CRY2

Samples of 12-day old seedlings were harvested 12 h after dawn of day 12 after sowing and seedlings were grown under long days (16 h light / 8 h darkness) photoperiodic conditions. Linear model methods (LiMMA) were used for determining differentially expressed genes (as reported in Mir et al., 2013).
